# Risk factors for periventricular-intraventricular haemorrhage severity in preterm infants: a propensity score-matched analysis

**DOI:** 10.1186/s12887-023-04114-x

**Published:** 2023-07-05

**Authors:** Jinglan Huang, Yan Wang, Tian Tian, Tingting Zhu, Jun Tang, Qian Gao, Tao Xiong

**Affiliations:** 1 Department of Pediatrics, West China Second University Hospital, Sichuan University, Chengdu, China; 2 Key Laboratory of Birth Defects and Related Diseases of Women and Children (Sichuan University), Ministry of Education, Chengdu, China; 3 Department of Neonatology, Maternal and Child Health Hospital of Zigong, Zigong, Sichuan 643000 China; 4 Department of Epidemiology and Biostatistics, School of Public Health, Institute of Reproductive and Child Health, Peking University Health Science Center, Beijing, China

**Keywords:** Preterm infants, Periventricular-intraventricular haemorrhage, Gestational age, Propensity score, Risk factor

## Abstract

**Background:**

Most previous studies comparing etiological studies in infants with and without periventricular-intraventricular haemorrhage (PV-IVH) concluded that younger gestational age (GA) was associated with a higher prevalence rate of PV-IVH. However, only a few studies have examined the risk factors associated with the severity of PV-IVH after removing the influence of GA. Therefore, we investigated the risk factors apart from GA for PV-IVH severity in preterm infants less than 28 weeks.

**Methods:**

This was a retrospective case-control study of preterm infants born in West China Second Hospital with PV-IVH between 2009 and 2020. PV-IVH was defined using cranial ultrasound screening. Preterm infants were divided into no PV-IVH and PV-IVH groups, and preterm infants with PV-IVH were divided into mild and severe PV-IVH groups. Groups were matched in a 1:1 ratio using propensity score calculated from GA. Variables were collected from infant–mother pairs. A stepwise forward multivariate logistic regression model was adopted to select factors that affected PV-IVH in preterm infants.

**Results:**

A total of 429 preterm infants were included. The total incidence of PV-IVH in preterm infants was 55.6%, and the incidence of mild and severe PV-IVH was 28.7% and 26.9%, respectively. We matched 162 infants with no PV-IVH with 162 infants with PV-IVH. The results suggested that electrolyte disorder (OR 2.79, 95% CI: 1.34–5.77), early-onset sepsis (OR 1.76, 95% CI: 1.01–3.08), thrombocytopenia (OR 2.87, 95% CI: 1.10–7.48), invasive mechanical ventilation (OR 4.21, 95% CI: 1.86–9.55), and male sex (OR 2.16, 95% CI: 1.29–3.60) were independently associated with PV-IVH. Then, we matched 87 infants with mild PV-IVH with 87 infants with severe PV-IVH. The results suggested that electrolyte disorder (OR 2.88, 95% CI: 1.29–6.45), thrombocytopenia (OR 5.73, 95% CI: 1.91–17.14), and invasive mechanical ventilation (OR 10.54, 95% CI: 1.16–95.85) were independently associated with severity of PV-IVH.

**Conclusions:**

Regardless of GA, electrolyte disorder, early-onset sepsis, thrombocytopenia, invasive mechanical ventilation, and male sex contributed to PV-IVH in preterm infants, and electrolyte disorder, thrombocytopenia, and invasive mechanical ventilation contributed to severe PV-IVH. These risk factors may combine to predict the incidence of PV-IVH in preterm infants.

**Supplementary Information:**

The online version contains supplementary material available at 10.1186/s12887-023-04114-x.

## Background

Premature births and their consequences pose significant clinical and social problems. Periventricular-intraventricular haemorrhage (PV-IVH) is a typical intracranial lesion in preterm infants [1], and the prognosis is mainly dependent on its severity [2]. A previous study suggests that preterm infants with mild PV-IVH are at a higher risk of cerebral palsy compared to preterm infants without PV-IVH [2]. As the grade of PV-IVH increases, the degree of neurological damage also increases; consequently severe PV-IVH (grades III and IV) is an independent risk factor for death and neurological sequelae [2, 3]. Survivors of severe PV-IVH often experience cognitive, language, and motor deficits, requiring extensive and early rehabilitation interventions to improve neurological outcomes [4]. Severe PV-IVH results in a heavy burden on the affected infants, their families, and society. Therefore, identifying the risk factors for different degrees of PV-IVH is essential and may facilitate a better understanding of the PV-IVH aetiology.

PV-IVH occurrence, particularly severe PV-IVH, is inversely related to gestational age (GA) [5–7]. The reasons include poor coagulation, fragile microvasculature in the germinal matrix, lack of autoregulatory mechanisms in the cerebral blood flow, and increased complications and treatments associated with the small size of preterm infants [8]. GA, the most influential and immutable factor, has been considered routinely in previous PV-IVH studies, which may have eliminated the effects of other factors. Therefore, exploring the other adjustable factors for PV-IVH is essential. Furthermore, current studies have only compared infants with and without PV-IVH in aetiology research [9, 10], only a few studies have examined the different risk factors between severe and mild PV-IVH after removing the influence of GA [11, 12]. Therefore, using propensity score matching for GA, this study was performed to explore the different risk factors for PV-IVH and the severity of PV-IVH.

## Materials and methods

### Study population

West China Second Hospital is a crucial tertiary hospital in Southwest China, where numerous critically ill newborns are treated in the neonatal intensive care unit. Our medical centre has 200 beds, including 100 cots in the neonatal intensive care unit and 100 cots in the general ward. Approximately 4,000 preterm infants are admitted to our centre each year. Per the American Academy of Neurology practice parameters and Chinese practice guidelines [13, 14], a cranial ultrasound is performed on all preterm infants admitted to our neonatal intensive care unit. In this study, we selected inborn preterm infants at less than 28 weeks of gestation between 2009 and 2020 to investigate the aetiologic of PV-IVH and the differences between severe and mild cases. Infants were excluded in the study if they were outborn with a severe deformity, genetic or metabolic disease, chromosome abnormality, or incomplete information.

### Diagnosis of PV-IVH

#### PV-IVH grading and grouping

We referred to the Papile grading system as follows [15]: grade I: haemorrhage restricted to the subependymal; grade II: intraventricular haemorrhage without ventricular dilatation; grade III: extended haemorrhage with ventricular dilatation; and grade IV: haemorrhage within the ventricular system and parenchymal. Grades I and II were regarded as mild PV-IVH, and grades III and IV were regarded as severe PV-IVH. If the highest PV-IVH grade detected on the serial cranial ultrasound was grade III or IV, the infants were included in the severe PV-IVH group. Otherwise, infants with grades I or II were included in the control group.

#### Cranial ultrasound screening

Serial cranial ultrasound was performed by paediatric sonographers as described in our previous study [8]. The initial screening was completed within 72 h after birth. If no bleeding was detected, the screening was repeated at 7, 14, and 42 days. If bleeding was observed, then a repeat ultrasound was performed weekly until at least two consecutive scans demonstrated stabilisation or resolution of bleeding. The paediatric sonographers received uniform PV-IVH screening training and were blinded to the neonates’ clinical information to ensure consistent and reliable reporting.

### Data collection

This study was a retrospective case-control study, and data were obtained from the patient database of our medical centre. We selected the possible risk factors for this study after reviewing numerous relevant studies on the risk factors of PV-IVH. All methods in this study adhered to the applicable guidelines and regulations.

The data on variables collected in this study were consistent with those in our previous study [8] and are described as follows: (1) infant baseline characteristics including GA, birth weight (BW), sex, multiple gestations, primigravidity, vaginal delivery, in vitro fertilisation, and asphyxia (umbilical artery pH < 7.0); (2) complications including pneumonia, respiratory distress syndrome, apnea (i.e., respiratory arrest for more than 20 s), patent ductus arteriosus, sclerederma, anaemia (i.e., venous haemoglobin < 130 g/L or peripheral blood haemoglobin < 145 g/L), and early-onset sepsis (onset at ≤ 3 days of age); (3) laboratory test results including electrolyte disorder (any abnormalities in electrolytes such as sodium ions, potassium ions, calcium ions, phosphate ions, chloride ions, and magnesium ions detected through laboratory tests), thrombocytopenia (platelet count ≤ 100 × 10^9^/L), and abnormal coagulation (i.e., activated partial thromboplastin time > 70 s); (4) treatments received including invasive mechanical ventilation (a history of receiving conventional ventilation or high-frequency oscillatory ventilation after endotracheal intubation), and administration of pulmonary surfactant, dopamine, or antibiotics; and (5) maternal characteristics including a history of foetal abnormalities (e.g., premature birth, teras, or hydatidiform mole), gestational hypertension, and histological chorioamnionitis. The history also included placental abnormality (e.g., placenta previa or placental abruption), anaemia (i.e., blood haemoglobin < 100 g/L), and intrahepatic cholestasis of pregnancy (ICP). Abnormal foetal position, lower genital tract infection (i.e., culture-positive vaginal and cervical secretions), foetal intrauterine distress, and use of antibiotics and dexamethasone (complete and incomplete course) were considered as part of the maternal history. Cases with incomplete data were not included in the final analysis.

### Statistical analysis

We used SPSS software (version 26, IBM Corp., Armonk, NY, USA) to perform the statistical analyses. First, infants were matched in a 1:1 ratio using the propensity score calculated from the GA. The propensity score was calculated by fitting a logistic regression model. Nearest-neighbor matching within a specified calliper width (a calliper of 0.2 standard deviations of the logit function of the propensity scores) was used. The first randomly selected infant in the PV-IVH group was matched to the patient in the no PV-IVH group with the closest propensity score. If multiple infants in the no PV-IVH group were equally close to this infant in the PV-IVH group, then one of the infants in the no PV-IVH group was randomly selected for matching with this infant treated in the PV-IVH group. This process was repeated until all possible matches were formed. If for a given infant in the PV-IVH group, no available patient in the no PV-IVH group was within the specified calliper width, then that infant in the PV-IVH group was excluded from the matched sample. Similarly, unmatched infants in the no PV-IVH group were excluded from the matched sample. The same method was used to match the mild and severe PV-IVH groups. Then, the risk factors for PV-IVH were evaluated between the mild and severe PV-IVH groups.

The continuous variables are presented as the mean ± standard deviation for normally distributed data, and independent sample t-tests were performed. The Wilcoxon test was used for continuous variables with a skewed distribution, which are presented as medians with interquartile ranges. Chi-square or Fisher’s exact tests were used to compare categorical variables.

Correlation matrix analysis and Chi-square test were performed to determine the correlation between two variables. For variables with correlation coefficients greater than 0.4, or Chi-square test with statistical differences, variables were selectively excluded. Finally, the variables that differed significantly between groups and without significant correlations were included in the multivariate analysis. Potential variables were used in a backward stepwise logistic regression analysis to calculate the adjusted odds ratios (ORs) and corresponding 95% confidence intervals (CIs). All hypothesis tests were two-tailed. Statistical significance was set at P < 0.05.

## Results

**Fig. 1:**
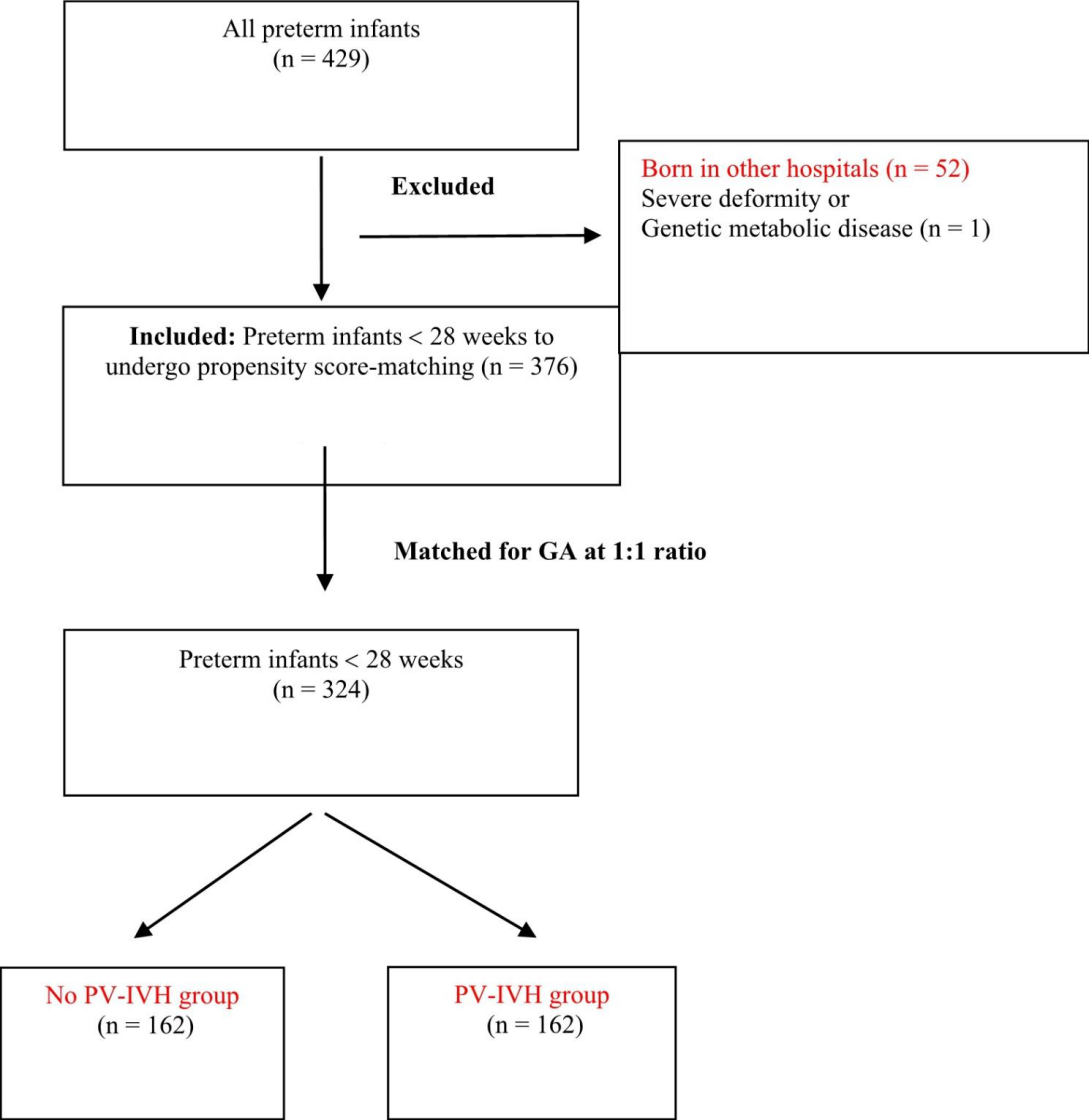
Flow Chart of the selection of preterm infants. *PV–IVH* periventricular–intraventricular haemorrhage

A total of 429 inborn premature infants (in West China Second Hospital) were included between 2009 and 2020. The total incidence of PV-IVH in preterm infants was 55.6%, and the incidence of mild and severe PV-IVH was 28.7% and 26.9%, respectively. Ultimately, 376 neonates at less than 28 weeks of gestation were included in this study. After propensity score-matching, 324 premature infants were used for analysis: 162 with no PV-IVH and 162 with PV-IVH (Fig. 1). Almost all of the PV-IVH preterm infants were diagnosed within 7 days of birth, and all the variables in our study were collected before PV-IVH onset. GA and BW presented a skewed distribution, with a median GA of 26.2 (23–27) weeks and median BW of 925 (440–1440) g and 940 (490–1400) g for the two groups, respectively. The groups comprised 176 boys and 148 girls.

**Fig. 2:**
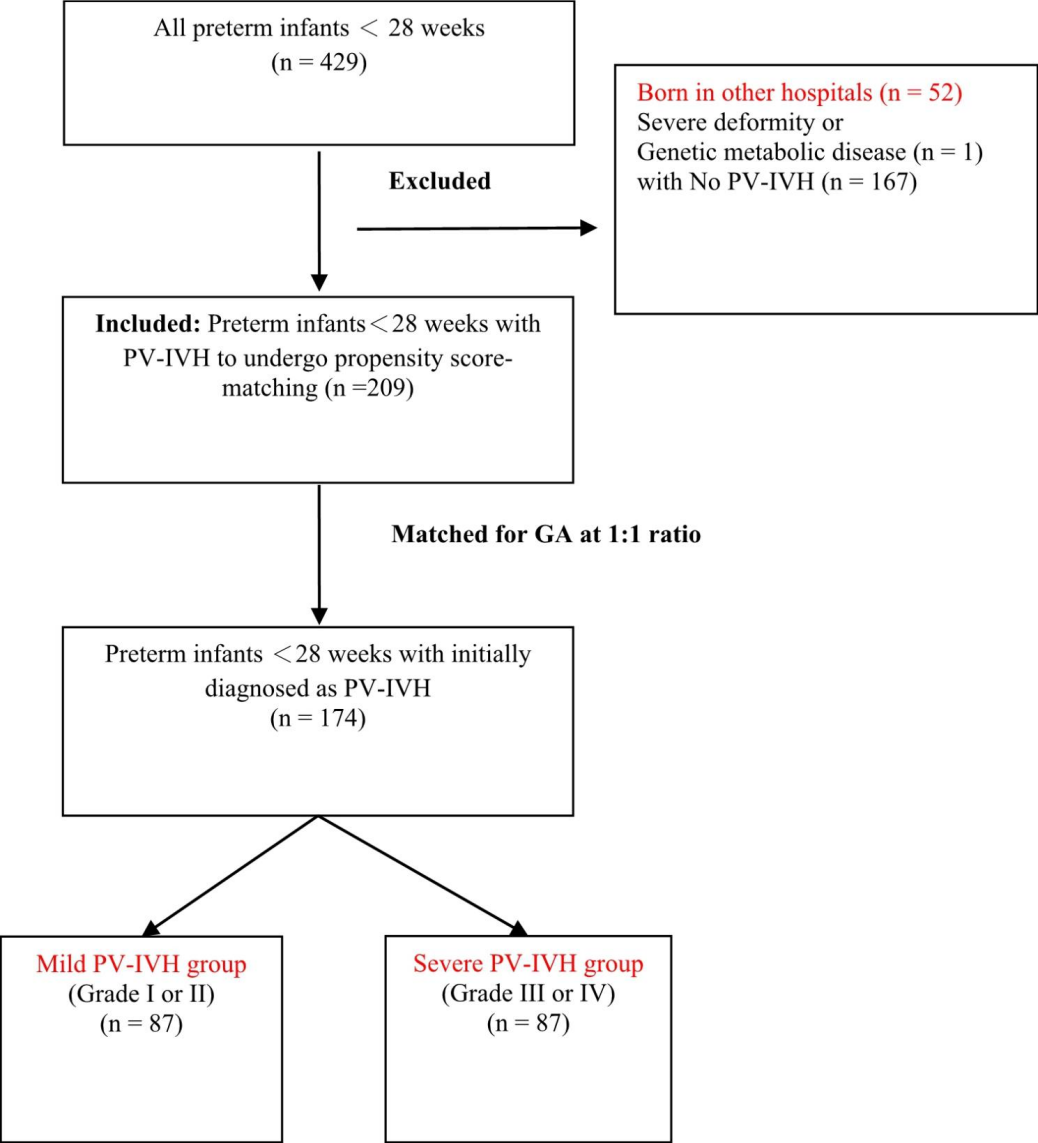
Flow Chart of the selection of preterm infants with PV-IVH. *PV–IVH* periventricular–intraventricular haemorrhage

Subsequently, after propensity score-matching, 174 premature infants with PV-IVH were used for analysis: 87 with mild PV-IVH and 87 with severe PV-IVH (Fig. 2).

General information regarding preterm infants with PV-IVH is presented in Table 1. We found no significant differences in GA, BW, primigravidity, multiple gestation, in vitro fertilisation, placental abruption, and asphyxia between the groups. Patients in the PV-IVH group were more likely to be male and by a vaginal delivery.

**Table 1:** Univariate analysis of the basic information of preterm infants after propensity score-matched pairs

Variables	No PV-IVH group (n = 162)	PV-IVH group (n = 162)	P
Gestational age median (IQR) — wk	26.2 (23–27)	26.2 (23–27)	1.000
Birth weight median (IQR) — g	925(440–1440)	940 (490–1400)	0.614
Male n, (%)	79 (48.8)	97 (59.9)	0.045
Primigravidity n, (%)	66 (40.7)	76 (46.9)	0.263
Multiple gestations n, (%)	79 (48.8)	84 (51.9)	0.579
Vaginal delivery n, (%)	38 (23.5)	58 (35.8)	0.015
In vitro fertilization n, (%)	11 (6.8)	15 (9.3)	0.413
Placental abruption n, (%)	21 (12.9)	21 (12.9)	1.000
Asphyxia n, (%)	151 (93.2)	146 (90.1)	0.315

IQR interquartile range; *PV–IVH* periventricular–intraventricular hemorrhage.

Table 2 shows the complications and laboratory tests of preterm infants. The preterm infants with sclerederma, early-onset sepsis, electrolyte disorder, and thrombocytopenia were more likely to have PV-IVH. There were no significant differences between the no PV-IVH and PV-IVH groups in terms of the incidence of pneumonia, respiratory distress syndrome, apnea, patent ductus arteriosus, anemia, and abnormal coagulation.

**Table 2:** Univariate analysis of preterm infant complications

Variables	No PV-IVH group (n = 162)	PV-IVH group (n = 162)	P
Pneumonia n, (%)	120 (74.1)	126 (77.8)	0.436
Respiratory distress syndrome n, (%)	147 (90.7)	146 (90.1)	0.850
Apnea n, (%)	43 (26.5)	52 (32.1)	0.272
Patent ductus arteriosus n, (%)	99 (61.1)	104 (64.2)	0.566
Scleredema n, (%)	4 (2.5)	22 (13.6)	< 0.001
Anemia n, (%)	95 (58.6)	95 (58.6)	1.000
Early-onset sepsis n, (%)	39 (24.1)	61 (37.7)	0.008
Electrolyte disorder n, (%)	16(9.9)	39(24.1)	0.001
Thrombocytopenia n, (%)	9 (5.6)	27 (16.7)	0.001
Abnormal coagulation n, (%)	15 (9.3)	22 (13.6)	0.221

*PV–IVH* periventricular–intraventricular haemorrhage. aBaseline values were tested before or within PV–IVH diagnosis.

Treatments administered to preterm infants are shown in Table 3. Our results showed that infants with PV-IVH required more invasive mechanical ventilation. The use of dopamine, pulmonary surfactant, and antibiotics did not differ between groups.

**Table 3:** Univariate analysis of the treatment of preterm infants

Variables	No PV-IVH group (n = 162)	PV-IVH group (n = 162)	P
Invasive mechanical ventilation n, (%)	125 (77.2)	151 (93.2)	< 0.001
Pulmonary surfactant use n, (%)	136 (84.0)	146 (90.1)	0.098
Dopamine n, (%)	18 (11.1)	25 (15.4)	0.252
Antibiotics n, (%)	120 (74.1)	127 (78.4)	0.361

*PV–IVH* periventricular–intraventricular hemorrhage.

Table 4 shows the maternal characteristics of preterm infants. We found that PV-IVH was associated with a maternal history of cesarean section, gestational hypertension, intrahepatic cholestasis of pregnancy, and lower genital tract infection. There were no significant differences between the no PV-IVH and PV-IVH groups in terms of the history of fetal abnormalities and abortion, pre-eclampsia, gestational diabetes, pregnancy anemia, chorioamnionitis, pregnancy with thrombocytopenia, and use of antibiotics and dexamethasone.

**Table 4:** Univariate analysis of maternal characteristics of premature infants

Variables	No PV-IVH group (n = 162)	PV-IVH group (n = 162)	P
History of fetal abnormalities n, (%)	8 (4.9)	10 (6.2)	0.566
History of abortion n, (%)	55 (34.0)	64(39.5)	0.162
History of cesarean section n, (%)	18 (11.1)	34 (21.0)	0.013
Pre-eclampsia n, (%)	6(3.7)	6(3.7)	0.991
Gestational hypertension n, (%)	3 (1.9)	14 (8.6)	0.006
Gestational diabetes n, (%)	36(22.2)	34(21.0)	0.833
ICP n, (%)	1 (0.6)	8 (4.9)	0.017
Pregnancy anemia n, (%)	26 (16.0)	32 (19.8)	0.382
Chorioamnionitis n, (%)	90(55.6)	91(56.2)	0.806
Lower genital tract infections n, (%)	35 (21.6)	62 (38.3)	0.001
Antibiotics n, (%)	94(58.0)	77(47.5)	0.262
Thrombocytopenia n, (%)	1(0.6)	2(1.2)	0.562
Dexamethasone n, (%)	137(84.6)	111(68.5)	0.112

*PV–IVH* periventricular–intraventricular hemorrhage, *ICP* intrahepatic cholestasis of pregnancy.

In the univariate analysis, 10 significant variables were obtained and included in the multivariate analysis after stepwise elimination, correlation coefficients, and clinical screening. The results (Table 5) suggested that early-onset sepsis (OR 1.76, 95% CI: 1.01–3.08), thrombocytopenia (OR 2.87, 95% CI: 1.10–7.48), electrolyte disorder (OR 2.79, 95% CI: 1.34–5.77), invasive mechanical ventilation (OR 4.21, 95% CI: 1.86–9.55), and male sex (OR 2.16, 95% CI: 1.29–3.60) were independently associated with PV-IVH.

**Table 5:** Factors associated with PV-IVH in preterm infants

Variables	aOR	95% CI
Early-onset sepsis	1.76	1.01–3.08
Thrombocytopenia	2.87	1.10–7.48
Electrolyte disorder*	2.79	1.34–5.77
Invasive mechanical ventilation	4.21	1.86–9.55
Male	2.16	1.29–3.60

*PV–IVH* periventricular–intraventricular haemorrhage, *aOR* adjusted odds ratio, *95% CI* 95% confidence interval. * refers to any abnormalities in electrolytes such as sodium ions, potassium ions, calcium ions, phosphate ions, chloride ions, and magnesium ions detected through laboratory tests

Table 6 shows the univariate analysis of preterm infants with PV-IVH. The preterm infants with early-onset sepsis, electrolyte disorder, thrombocytopenia, and invasive mechanical ventilation were more likely to have severe PV-IVH than mild PV-IVH. There were no significant differences in male sex between the mild and severe PV-IVH groups.

**Table 6:** Univariate analysis of premature infants with PV-IVH.

Variables	Mild PV-IVH group (n = 87)	Severe PV-IVH group (n = 87)	P
Male n, (%)	50 (57.5)	44 (50.6)	0.361
Early-onset sepsis n, (%)	24 (27.6)	41 (47.1)	0.008
Electrolyte disorder n, (%)	12 (13.8)	29 (33.3)	0.002
Thrombocytopenia n, (%)	5 (5.7)	26 (29.9)	< 0.001
Invasive mechanical ventilation n, (%)	78 (89.7)	86 (98.9)	0.009

*PV–IVH* periventricular–intraventricular hemorrhage.

The results (Table 7) suggested that electrolyte disorder (OR 2.88, 95% CI: 1.29–6.45), thrombocytopenia (OR 5.73, 95% CI: 1.91–17.14), and invasive mechanical ventilation (OR 10.54, 95% CI: 1.16–95.85) were independently associated with severity of PV-IVH.

**Table 7:** Factors associated with severity of PV-IVH in preterm infants

Variables	aOR	95% CI
Thrombocytopenia	5.73	1.91–17.14
Electrolyte disorder	2.88	1.29–6.45
Invasive mechanical ventilation	10.54	1.16–95.85

*PV–IVH* periventricular–intraventricular haemorrhage, *aOR* adjusted odds ratio, *95% CI* 95% confidence interval.

## Discussion

The most influential risk factor for PV-IVH, GA is inversely correlated with the occurrence of PV-IVH [6, 8]. In this study, we conducted a propensity score-matched analysis to eliminate the influence of GA. Our study helps clarify the aetioly of PV-IVH, and the differences between the degrees of PV-IVH severity, which may be helpful for future clinical interventions.

In all premature infants included in this study, PV-IVH was diagnosed within 7 days of birth, and all risk factors occurred before the diagnosis of PV-IVH. Therefore, it is reasonable to believe that the risk factors we obtained may lead to PV-IVH, given that these risk factors have also been discussed in previous studies.

Our research shows that early-onset sepsis is a significant cause of PV-IVH, consistent with previous studies [5, 8, 11, 16]. Sepsis is known to be accompanied by a storm of inflammatory cytokines. Inflammatory factors detected in the blood of infants with PV-IVH were significantly higher than those in healthy neonates, correlating with PV-IVH severity [5, 8, 11, 16]. The increased level of inflammatory factors damage the blood-brain barrier and the cerebral blood vessels, contributing to the cerebral blood vessels’ rupture [17]. Inflammatory factors can also disrupt the blood pressure regulation function of the brain, resulting in severe fluctuations in cerebral perfusion pressure in premature infants and subsequent PV-IVH [10, 18].

A systematic review suggested that thrombocytopenia is a risk factor for PV-IVH in preterm infants [19], which is consistent with our findings. Since thrombocyte counts regulate haemostatic function, thrombocytopenia is associated with bleeding tendencies, including PV-IVH. In addition, thrombocytopenia may be caused by infection and other abnormal coagulation events [20, 21], which may be risk factors for PV-IVH.

Our study suggests that invasive mechanical ventilation is an independent risk factor for PV-IVH (Table 5). Previous studies have shown that invasive mechanical ventilation may disrupt cerebral blood flow and induces a local inflammatory response [17, 22]. Another study found that invasive mechanical ventilation aggravated brain damage by increasing dopamine release [23]. This provides a possible explanation for why premature infants are prone to PV-IVH after invasive mechanical ventilation.

We found that electrolyte disorder was an independent risk factor for PV-IVH and is related to its severity. Previous studies have suggested that a preterm infant’s immature brain is vulnerable to injury from an electrolyte disorder, such as hypernatremia, leading to changes in the osmolarity of intracranial blood vessels, which increases the risk of PV-IVH [9, 24, 25].

We found that male sex was an independent risk factor of PV-IVH, as supported by a previous study [26, 27]. The study suggests that male newborns are more susceptible to catecholamines and have a higher cerebral blood flow than female infants. In addition, sex differences in inflammatory factor gene polymorphisms and differences in hormone levels may explain why males are more prone to PV-IVH than females [26].

Our study had some limitations. First, we included preterm infants with a GA of less than 28 weeks. Hence, caution should be exercised when applying these findings to preterm infants with other GAs. Second, caution should be exercised when generalising our results to other settings, as this was a single-centre study conducted in China. In addition, there may be uncontrollable recall bias because this was a retrospective clinical study. Lastly, although the paediatric sonographers in this study received uniform PV-IVH screening training and were blinded to the clinical information, detection bias might have existed between paediatric sonographers.

## Conclusions

Our findings suggest that electrolyte disorder, early-onset sepsis, thrombocytopenia, invasive mechanical ventilation, and male sex contribute to PV-IVH in preterm infants. Also, electrolyte disorder, thrombocytopenia, and invasive mechanical ventilation contributed to severe PV-IVH.

## Electronic supplementary material

Below is the link to the electronic supplementary material.

Supplementary Material 1

Supplementary Material 2

## Data Availability

The data that support the findings of this study are available from West China Second University Hospital, but restrictions apply to the availability of these data, which were used under license for the current study, and so are not publicly available. Data are however available from the corresponding authors upon reasonable request and with permission of West China Second Hospital. Source data for PV-IVH after matching for GA are provided as Additional file 1.

## List of abbreviations

BW: Birth weight
CI: Confidence interval
GA: Gestational age
PV-IVH: Periventricular-intraventricular haemorrhage
ICP: Intrahepatic cholestasis of pregnancy
OR: Odds ratio
